# Supplementary material for: Structural and mechanistic insights into a lysosomal membrane enzyme HGSNAT involved in Sanfilippo syndrome
Source: Nat Commun. 2024 Jun 25;15:5388. doi: 10.1038/s41467-024-49614-1 (PMC11199644; doi:10.1038/s41467-024-49614-1)
Supplement: Supplementary file 1 — Supplementary Information [file 41467_2024_49614_MOESM1_ESM.pdf]

Supplementary information

Structural and mechanistic insights into a lysosomal membrane enzyme HGSNAT involved in Sanfilippo syndrome

Authors: Boyang Zhao[1§], Zhongzheng Cao[2§], Yi Zheng[3], Phuong Nguyen[4†], Alisa Bowen[4‡], Robert H. Edwards[5], Robert M. Stroud[4], Yi Zhou[2], Menno Van Lookeren Campagne[2], Fei Li[1*]

*Corresponding author. Email: fli05@amgen.com

This file contains:

• Supplementary Figure 1-9

• Supplementary Table 1

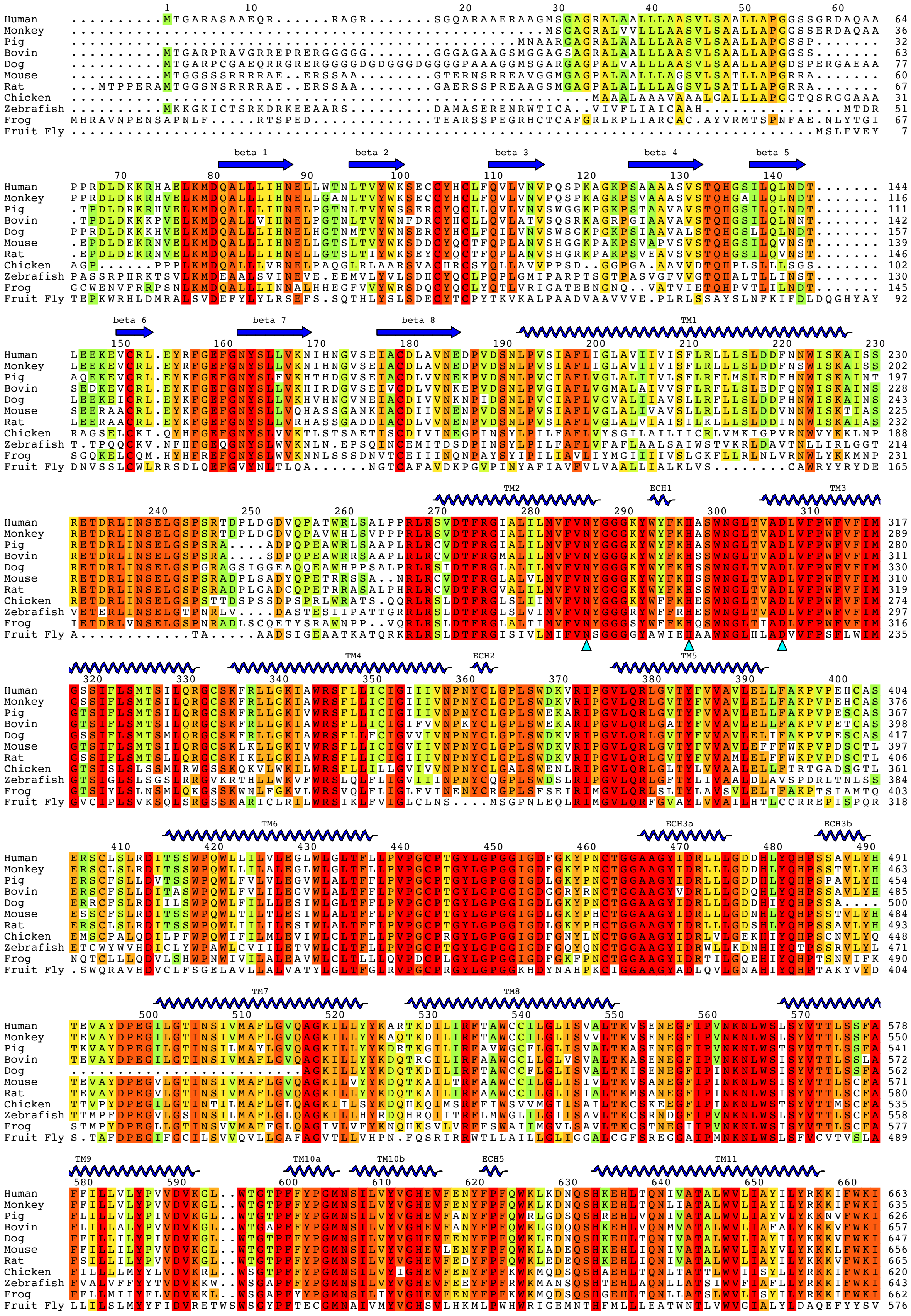
Human
Monkey
Pig
Bovin
Dog
Mouse
Rat
Chicken
Zebrafish
Frog
Fruit Fly
beta 1
beta 2
beta 3
beta 4
beta 5
beta 6
beta 7
beta 8
TM1
TM2
ECH1
TM3
TM4
ECH2
TM5
TM6
ECH3a
ECH3b
TM7
TM8
TM9
TM10a
TM10b
ECH5
TM11

Supplementary Fig. 1 | Sequence alignment of HGSNAT from representative species. Sequences of selected HGSNAT homologs. Residues are colored based on their sequence conservation, from most conserved (red) to least conserved (no color). The catalytic residues (N286, H297, and D307) are labeled with a cyan triangle. Figure is generated with ALINE[41].

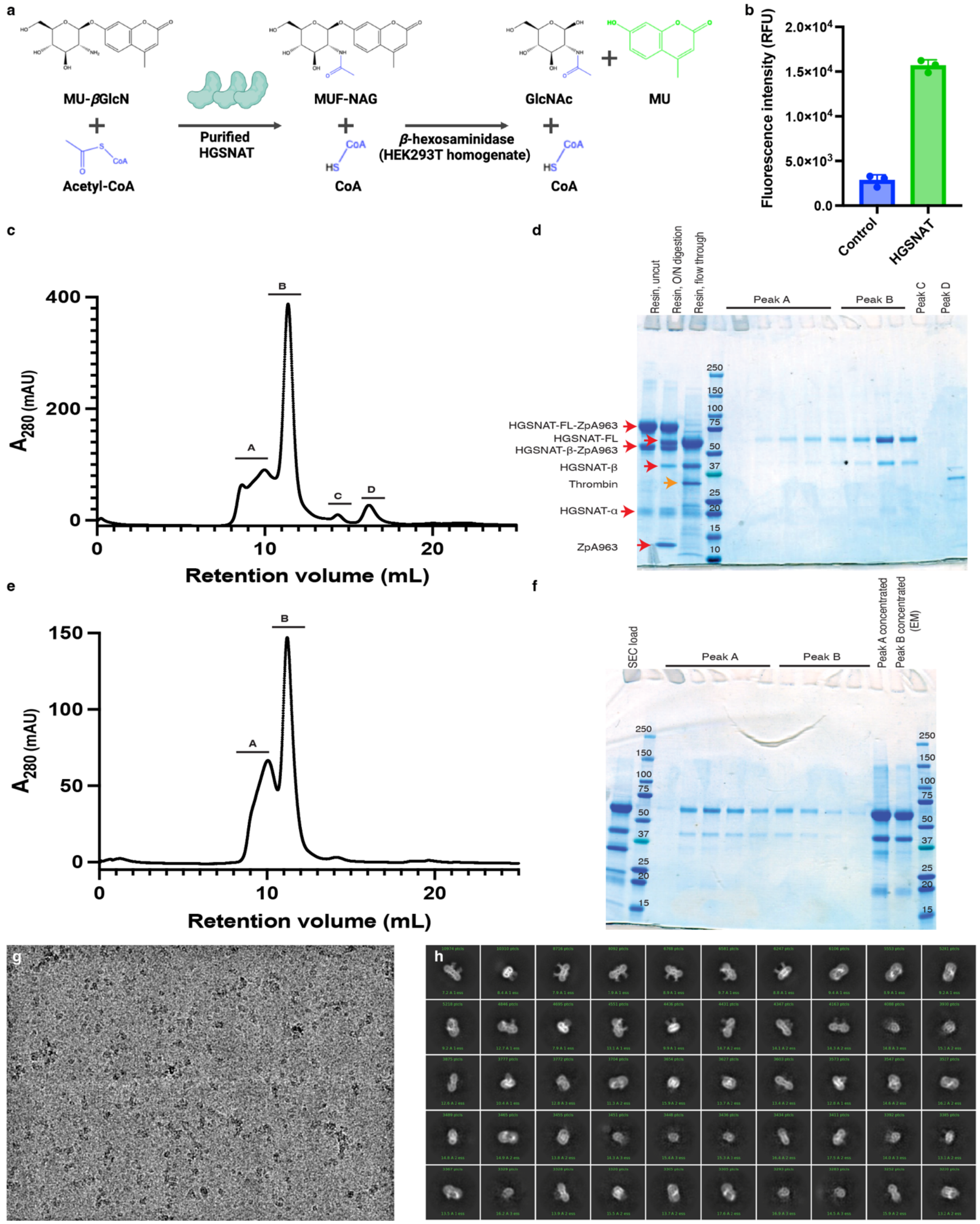

Supplementary Fig. 2 | Characterization of purified human HGSNAT used for cryo-EM. **a**, Schematic of HGSNAT enzymatic assay. **b**, Activity of purified HGSNAT is measured as fluorescence intensity of MU. HEK293T homogenate without addition of purified HGSNAT protein is used as control. Results are shown as mean ± SD (n = 3 replicates). Source data are

provided as a Source Data file. **c**, SEC of HGSNAT purified in DDM. **d**, SDS-PAGE of HGSNAT purified in DDM. **e**, SEC of HGSNAT in buffer containing GDN used for cryo-EM. **f**, SDS-PAGE of HGSNAT used for cryo-EM. **g**, Representative micrograph for HGSNAT. **h**, 2D classification of HGSNAT. Supplementary Figure 2a was created with BioRender.com released under a Creative Commons Attribution-NonCommercial-NoDerivs 4.0 International license https://creativecommons.org/licenses/by-nc-nd/4.0/deed.en.

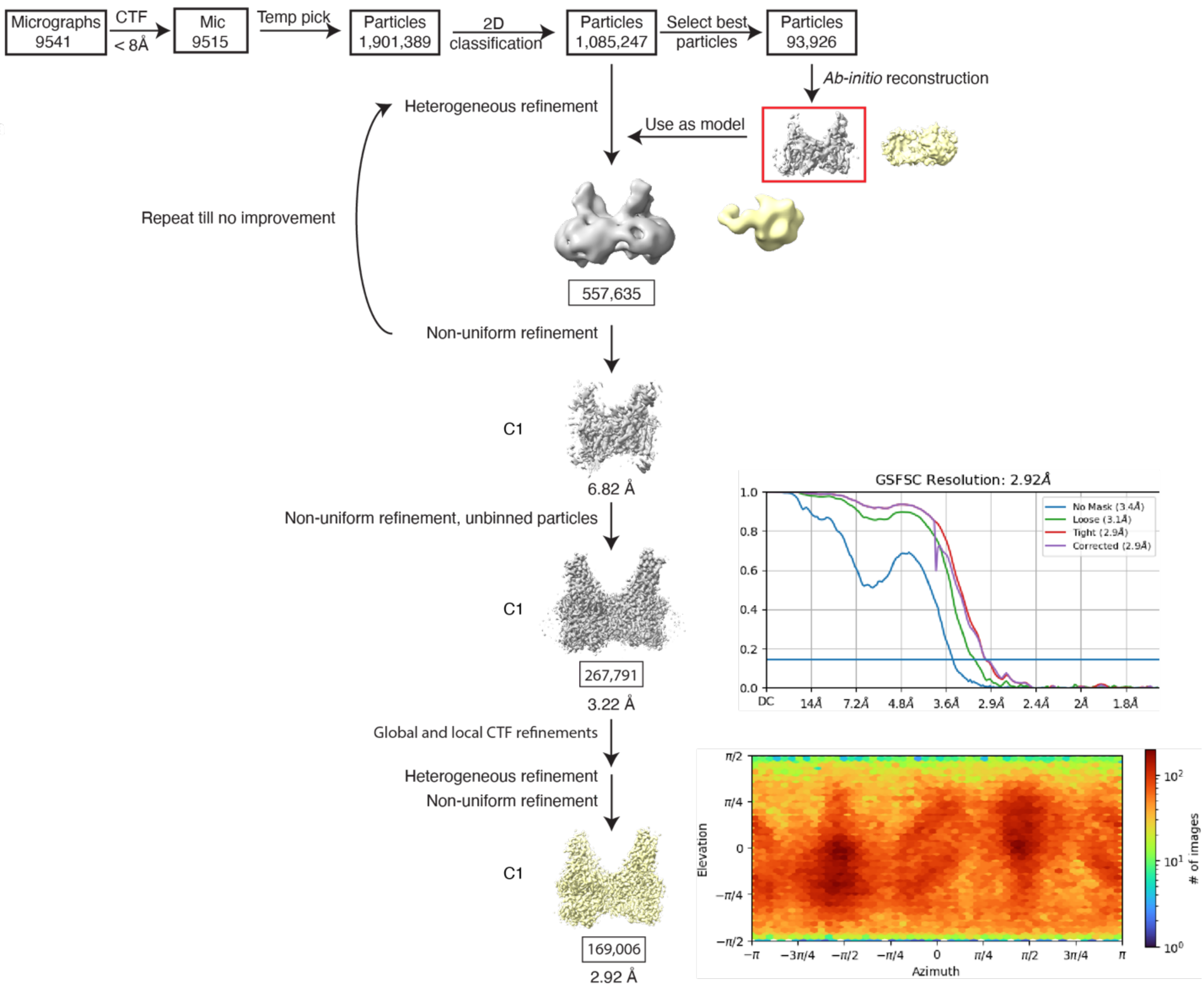

Supplementary Fig. 3 | Representative cryo-EM data processing workflow.
Data processing workflow for the Acetyl-CoA structure is shown as representative. Details are described in the methods section. All datasets are processed similarly, unless otherwise specified.

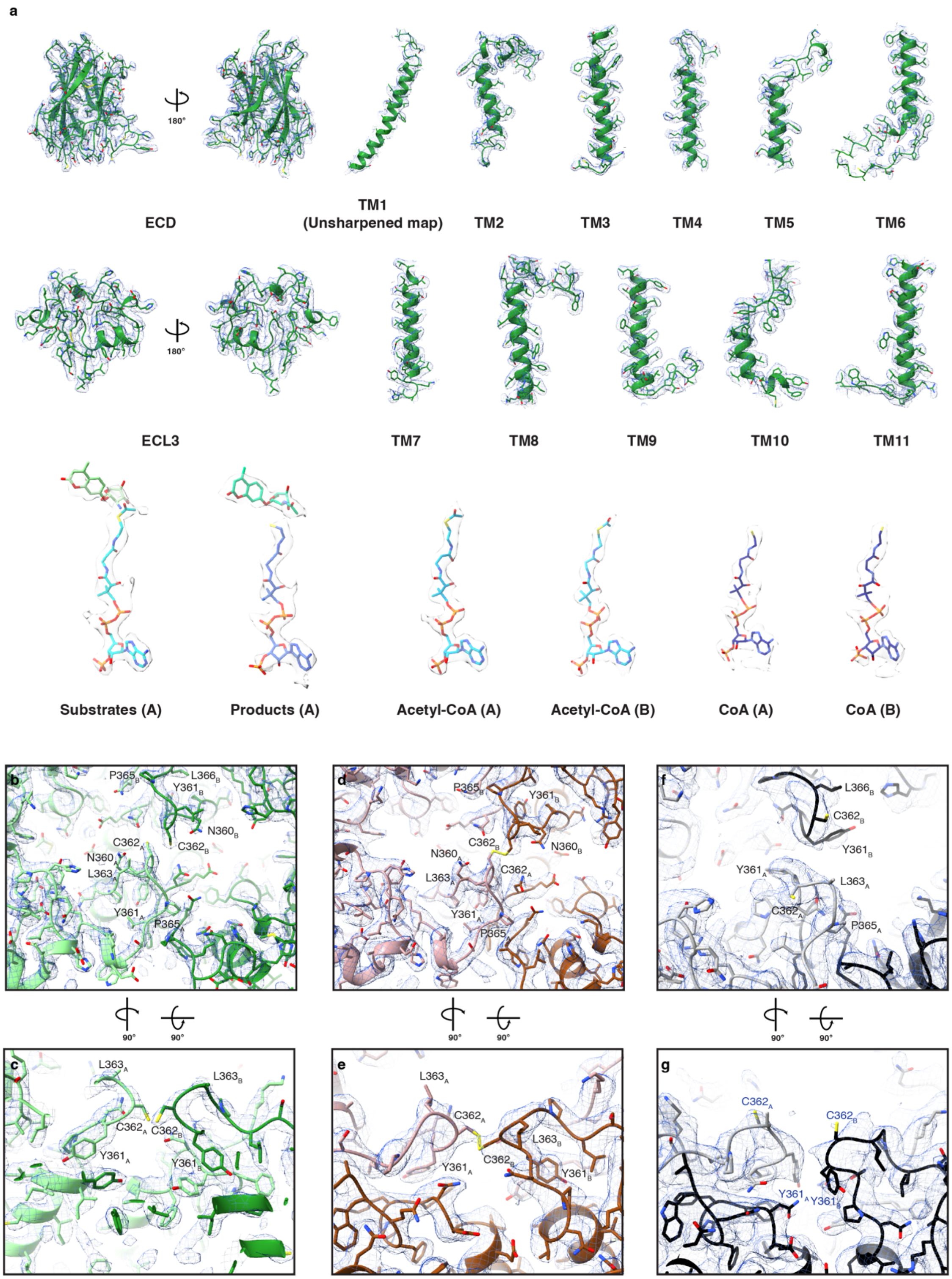

Supplementary Fig. 4 | Coulombic potential map for representative regions of structures of HGSNAT.

a, Coulombic potential map for HGSNAT protein in the Acetyl-CoA structure and densities for all ligands not shown in (Fig. 2). **b**, **c**, Density at the dimer interface for the Acetyl-CoA structure. **d, e**, Density at the dimer interface for the Apo$_{trans}$ structure. **f, g**, Density at the dimer interface for the Apo$_{ground}$ structure.

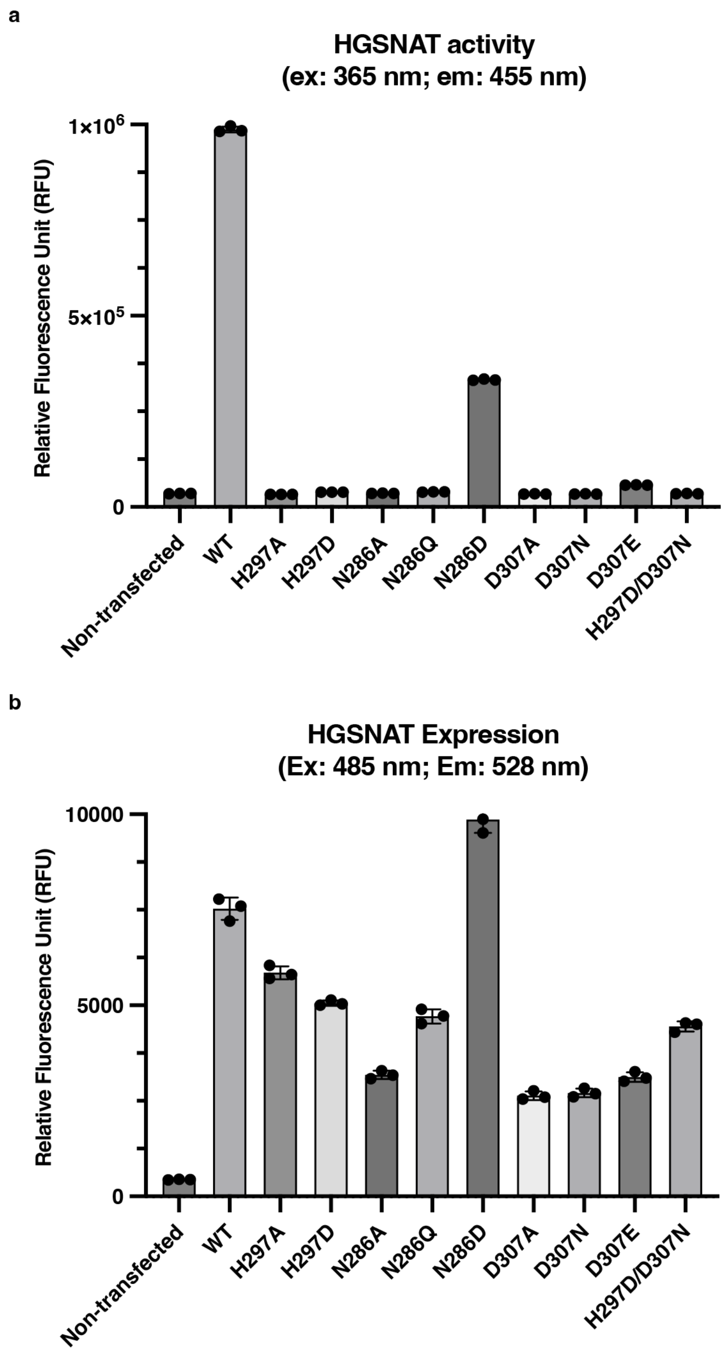

Supplementary Fig. 5 | Enzymatic activity of HGSNAT mutations at the active site.
a, Activity of HEK293T cell expressing HGSNAT-EGFP measured as release of fluorescence product β-Methylumbelliferone (4-MU). Results are shown as mean ± SD (n = 3 replicates). **b**, Expression level of HGSNAT-EGFP measured as GFP fluorescence. Results are shown as mean ± SD (n = 3 replicates). Source data are provided as a Source Data file.

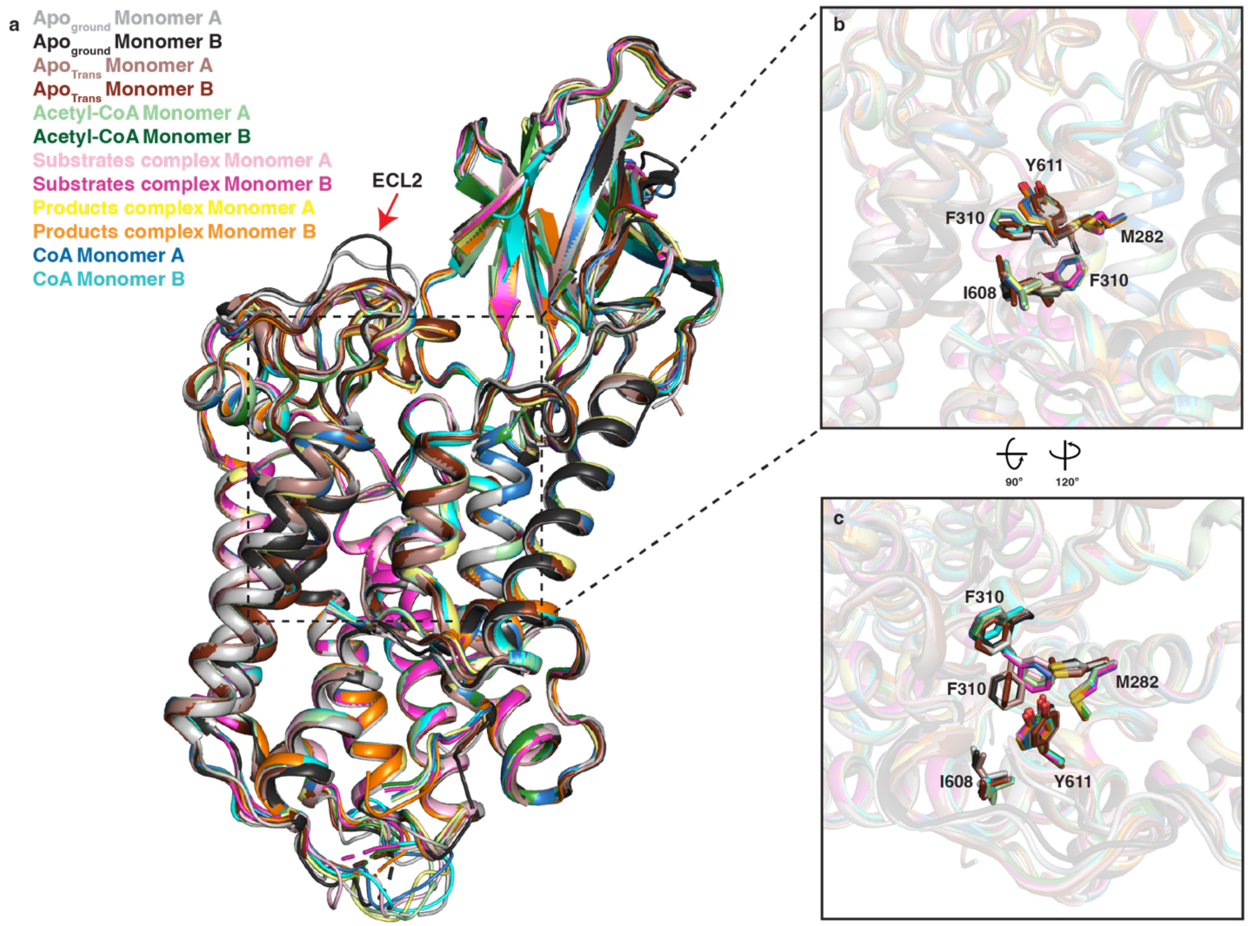

Supplementary Fig. 6 | Overlay of all monomers of HGSNAT from all 6 structures.
a, Overlay of all structures shows that there is no major conformational change of the TMD between different states. ECL2 exhibits a conformational change and influences the dimer formation. **b, c**, Zoom in at the acetyl-CoA gating residues.

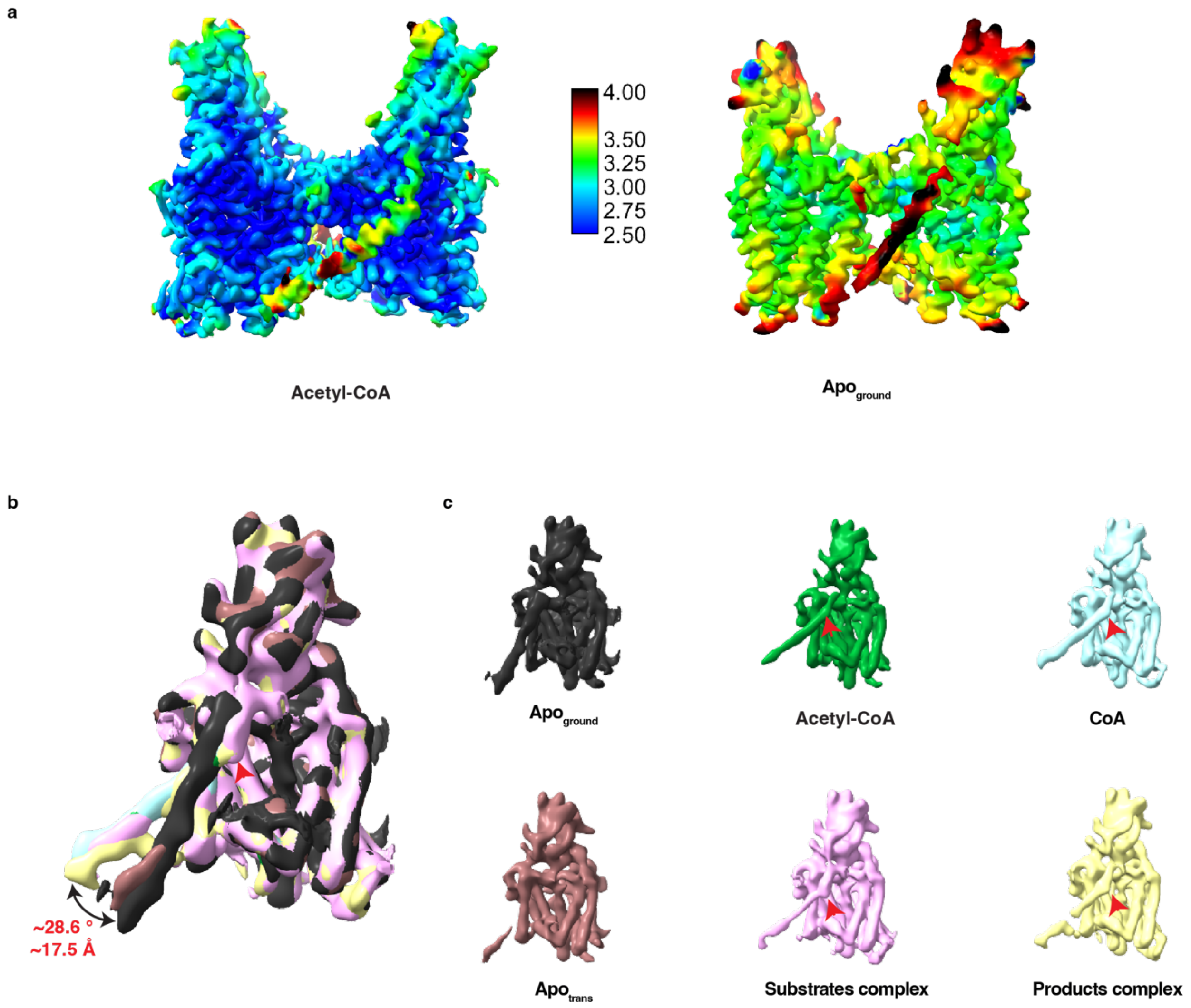

Supplementary Fig. 7 | TM1 is highly flexible.
a, Local resolution plots of unsharpened maps of the Acetyl-CoA structure and the Apo_{ground} structure show that TM1 is of much lower resolution than the rest of the protein. **b**, Overlay of maps of one monomer from all structures in different states low-pass filtered at 7 Å to demonstrate the range of motion of TM1. The N-terminal half of TM1 in the Apo_{trans} structure is invisible, while the C-terminal half is at a position similar to that of the TM1 in the Apo_{ground} structure. Position of the kink around I196 is marked with a red arrow. **c**, Individual maps shown in (**b**).

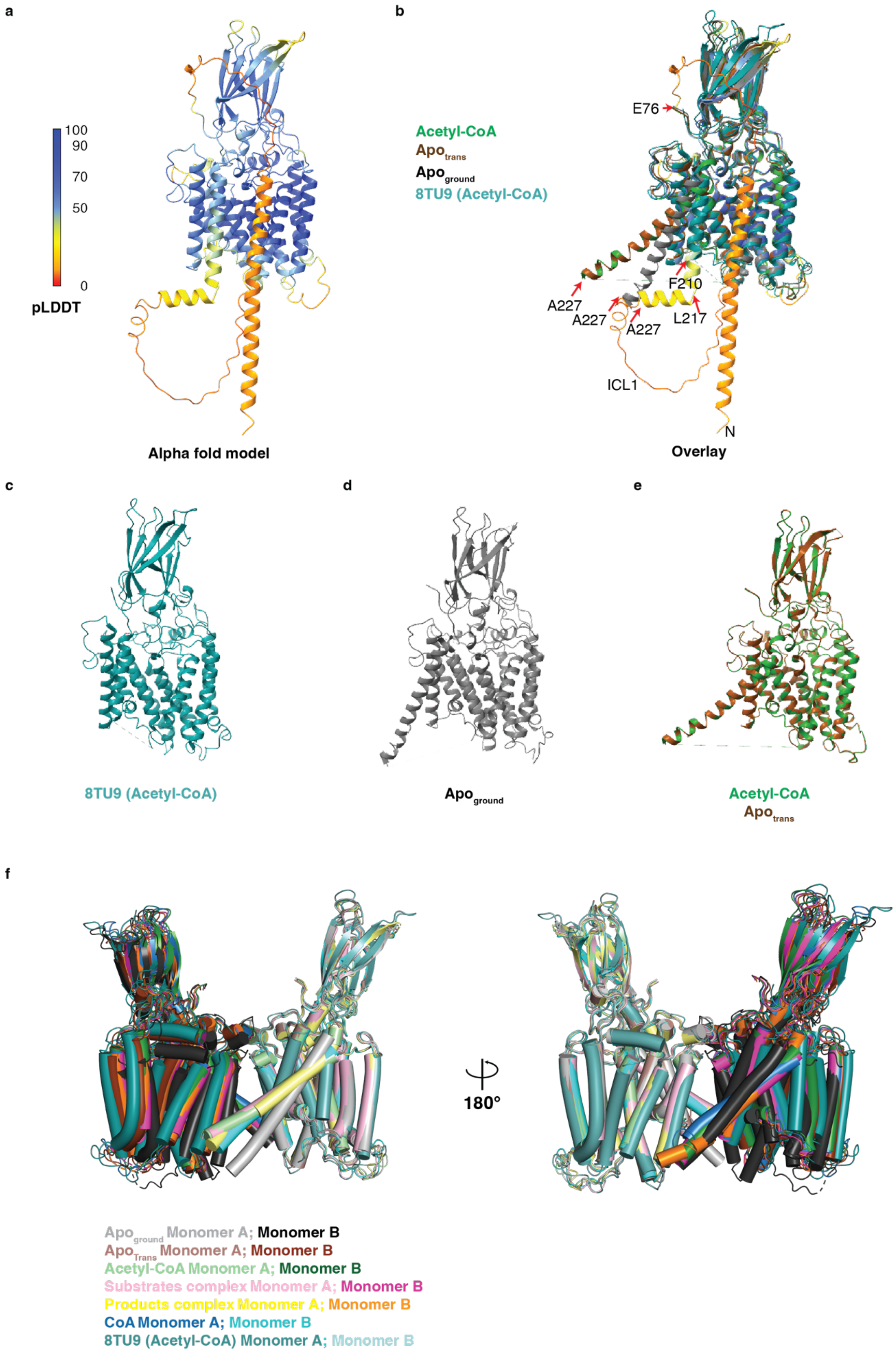
a
100
90
70
50
0
pLDDT
Alpha fold model
b
Acetyl-CoA
Apo$_{trans}$
Apo$_{ground}$
8TU9 (Acetyl-CoA)
E76
F210
A227
A227
A227
L217
ICL1
N
Overlay
c
8TU9 (Acetyl-CoA)
d
Apo$_{ground}$
e
Acetyl-CoA
Apo$_{trans}$
f
180°
Apo$_{ground}$ Monomer A; Monomer B
Apo$_{Trans}$ Monomer A; Monomer B
Acetyl-CoA Monomer A; Monomer B
Substrates complex Monomer A; Monomer B
Products complex Monomer A; Monomer B
CoA Monomer A; Monomer B
8TU9 (Acetyl-CoA) Monomer A; Monomer B

Supplementary Fig. 8 | Dynamics of HGSNAT.

a, AlphaFold model of HGSNAT monomer**. b,** Overlay of monomer of HGSNAT shows that TM1 is highly dynamic**. c,** structure of the monomer of HGSNAT bound with Acetyl-CoA determined by *Navratna et al* (8TU9). **d**, Structure of HGNSTA in Apo_{ground} state. **e,** Overlay of structure of HGSNAT in Apo_{trans} state and Acetyl-CoA bound state. **f**, Dimers of all 6 structures as well as 8TU9 are aligned over monomer A to show the large dynamics of the dimer assembly. Note, ligands are not shown in the structures.

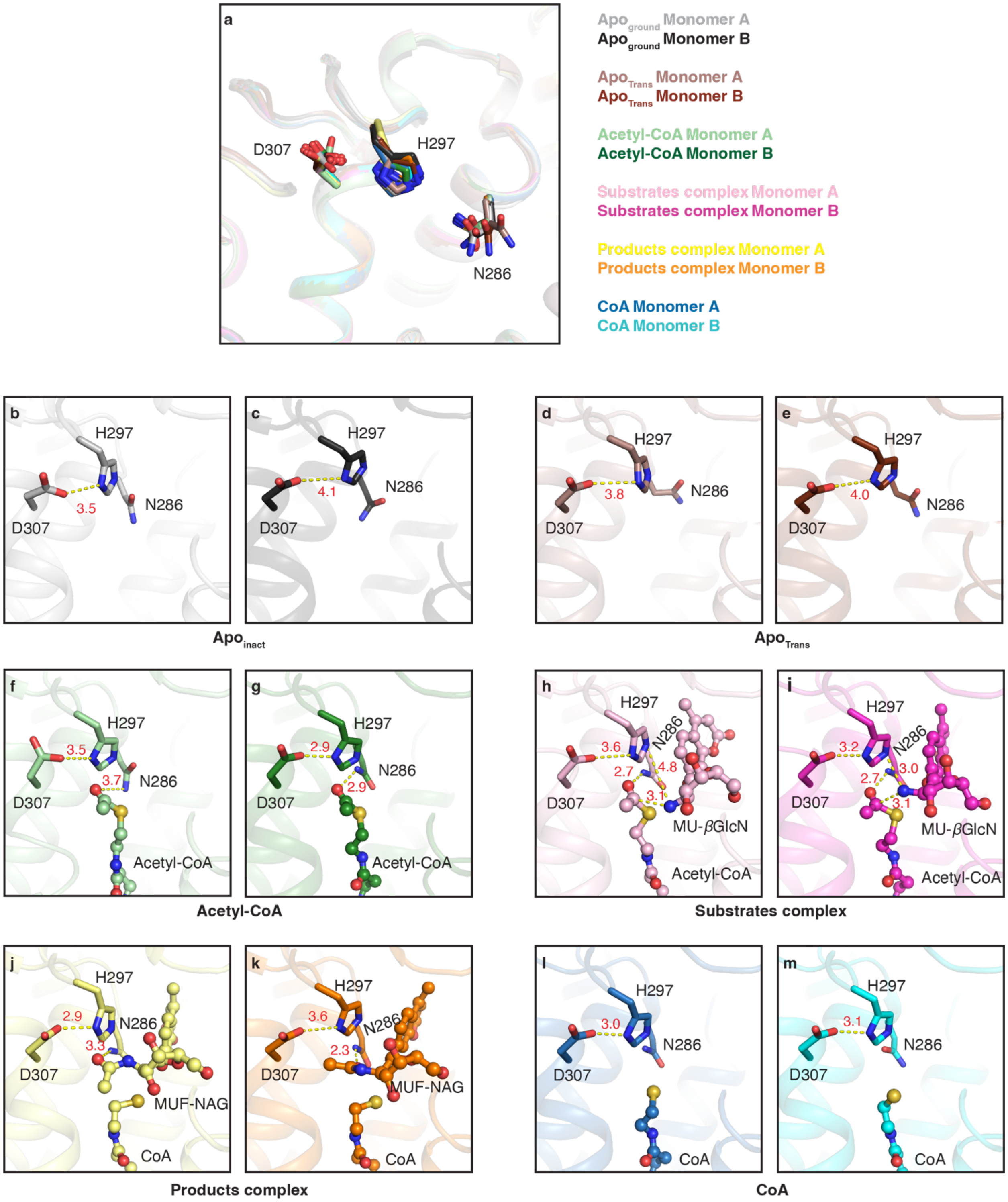

Supplementary Fig. 9 | Comparison of the active site structures.
a, Overlay of the active site of all monomers. **b-m**, Details of each structure shown in (**a**).

Supplementary table 1
Cryo-EM data collection, refinement and validation statistics

	Apo$_{ground}$ EMD-43345 8VLV	Apo$_{trans}$ EMD-43348 8VLY	Acetyl-CoA EMD-43319 8VKJ	Substrates complex EMD-43338 8VLG	Products complex EMD-43339 8VLI	CoA EMD-43344 8VLU
**Data collection and processing**						
Microscope	UCSF Titan Krios 2		Amgen/USC Titan Krios			
Camera	Gatan K3		Gatan K3			
Magnification	105000		105000			
GIF slit width (eV)	20		20			
Voltage (kV)	300		300			
Electron exposure (e^{-}/Å^{2})	43		56	50	50	52.7
Number of frames per Mic	80		50	50	50	50
Automation software	SerialEM		EPU			
Defocus range (μm)	1.0 - 2.2		1.0 – 2.2			
Pixel size (Å)	0.834		0.813			
Symmetry	C1		C1			
Number of movies	4824		9541	8019	7942	8898
Initial particle images	1,475,430		1,901,389	3,454,358	2,500,635	3,000,759
Refined particle images	488,511		1,085,247	541,888	2,410,255	2,583,324
Final particle images	134,353	226,631	169,006	202,590	276,382	256,492
Map resolution (Å)	3.49	3.61	2.92	3.15	3.20	3.12
FSC threshold	0.143	0.143	0.143	0.143	0.143	0.143
**Refinement**						
Initial model used	AF model		Apo$_{ground}$ structure (8VLV)			
Model resolution (Å)	3.7	3.7	3.0	3.3	3.3	3.2
FSC threshold	0.5	0.5	0.5	0.5	0.5	0.5
Model vs map CC	0.83	0.85	0.85	0.84	0.83	0.85
Model composition						
Non-hydrogen atoms	8269	7755	8622	8315	8446	8596
Protein residues	1085	992	1089	1089	1093	1096
Ligands	NAG: 9	NAG: 7	ACO:2 NAG: 7	ACO:2 NAG: 6	COA:2 MUG:2 NAG:7	COA:2 NAG:7
B factors (Å^{2})						
Protein	77.75	38.62	42.49	41.01	27.94	40.75
Ligand	103.68	45.06	37.79	38.38	59.84	51.58
R.m.s. deviations						
Bond lengths (Å)	0.003 (0)	0.003 (0)	0.003 (0)	0.004 (0)	0.003 (0)	0.004 (0)
Bond angles (°)	0.540 (2)	0.553 (1)	0.538 (2)	0.573 (1)	0.556 (1)	0.550 (0)
Validation						
MolProbity score	1.87	1.74	1.78	1.91	1.87	1.85
Clashscore	9.77	7.43	9.30	11.16	9.48	8.95
Poor rotamers (%)	0.12	0.0	0.00	0.00	0.00	0.12
CaBLAM outliers (%)	4.27	2.92	3.68	2.74	4.13	3.90
Cβ deviation	0	0	0	0	0	0
Ramachandran plot						
Favored (%)	94.86	95.29	95.81	94.97	94.62	94.66
Allowed (%)	5.14	4.71	4.09	5.03	5.38	5.34
Disallowed (%)	0.00	0.00	0.00	0.00	0.00	0.00

Reference:

41 Bond, C. S. & Schüttelkopf, A. W. ALINE: a WYSIWYG protein-sequence alignment editor for publication-quality alignments. *Acta Crystallogr D Biol Crystallogr* **65**, 510-512 (2009). https://doi.org/10.1107/S0907444909007835
